# Chemical Analysis of Rhubarb or Pie-plant

**Published:** 1845-08

**Authors:** E. R. Long

**Affiliations:** U. S. A.


*Chemical Analysis of Rhubarb or Pie-plant.*—Communicated for the *Buff. Med. Jour.* by Lieut. E. R. LONG, M.D., U. S. A.

DR. FLINT,—Dear Sir: In the first number of your journal, you notice a case of supposed poisoning from the Pie-plant, and request an analysis of the same, to determine the proportion of Oxalic Acid that enters into its composition, as this is presumed to be its deleterious principle.

I have submitted this vegetable to the process given below, and as the results in two experiments were the same, it is thought to be sufficiently accurate for all practical purposes.

Process:—Take $\frac{1}{4}$ lb. of the stalks (petioles) of the plant, reduce them to a pulp on a grater; add a pint of rain water; then bi-carb Potass. ℨij to separate the free Oxalic Acid from the other elements of the vegetable. Pass the liquor through a course filter, to remove the vegetable fibre and other insoluble ingredients. To the solution add mur. lime q. s. to precipitate the Oxalic Acid in the form of an insoluble Oxalate of Lime; collect this on a paper filter and dry it. We thus obtain all the free acid in the plant. To extract the portion which is in combination with lime, treat the residue found in the first filter with nitric acid; add bi-carb. Potass. and filter through paper as before; collect the matter on the filter and dry it. This is also Oxalate of Lime, soluble in Nitric Acid, and insoluble in vinegar, or acetic acid; subjecting these two portions of Oxalate of Lime to a red heat, it will be converted into carb. of lime, weight gr. 10. This gives sufficient data to ascertain the proportion of Oxalic Acid. For as Carb. lime consists of Ca. O. plus C. O2, 28,5—1 eq. base plus 22,12 1 eq. acid=50,62 eq., we have 28,5 parts of lime, in 50,62 parts of the Carb. There being 10 gr. of the latter, of course there will be 5,3 gr. of the former; for Oxalate of lime is composed of Ca. O. 28,5 plus C2 O3 36, plus 2 aq. 18=87,74, eq. From which we see that the lime is to the Oxalic acid in the ratio of 28,5 36. Hence, as the combining equivalents of the lime, in the Carb. and Oxalate are the same, if we have 5,3 gr. of lime, there will be 6 4-10 gr. of the Oxalic acid. This gives 24 3-5 gr. of the acid to 1 lb (avoirdupois) of the plant.

In the latter part of the above process we observe a beautiful exhibition of elective affinity. When the Nitric acid is added, the lime lets go the Oxalic acid and unites with the Nitric; but upon the addition of the Bi. Carb. Potass. the Nitric acid having a stronger affinity for the alkali than for the lime, gives up the latter and unites by preference to the former; when the Oxalic acid again reunites to the lime, to the exclusion of the Carb. acid, which is also present in a nascent state;—the most favorable for chemical union.

It was also remarked that the Oxalate of lime before it was exposed to heat, weighed 16 gr. and the Carb. produced 10

gr.; the eq. of the Oxalate being 82,74, and the eq. of the Carb. 50,62; it will appear that the equivalents and the weights are in the same ratio. This confirms the theory of the composition of the Oxalic acid, i. e. C. O. plus C. O2.; for the Oxalate losing one eq. of C. O. (14.) and two eq. of water (18); total loss 32: this deducted from its eq. 82,74 leave 50,74, the equivalent (nearly) of the Carb.

If the above analysis be correct is seems that the small bundles of the Pie-plant found in market, weighing about 1 lb., contain a little more than ℈j. of the acid. Now the question of practical importance is, whether any danger is to be apprehended from its use as an article of diet. The minimum fatal dose of the Crystalized acid on record in Standard Works, is ℥ss; but it would doubtless be unsafe to take a much smaller dose than this of the acid in a free state. Yet as the dilute acid is regarded and used as a safe refrigerant in fevers, and as a portion of it in the Pie-plant exists in combination with lime and is therefore inert, it would hardly seem probable that any deleterious effects would result from the ordinary use of the plant.

It may not be amiss to remark, that in a case of suspected poisoning from this acid, the proper antidote is Carbonate of lime, or Carb. of Magnesia, as these will form insoluble Salts with the acid.—The alkalies form soluble Oxalates possessing poisonous properties.

I will add that in one of the above experiments the Petioles or Stalks, were used; in the other, both the stalk and leaf, without any appreciable difference in the result.

Yours, truly,

*June 5th*, 1845. E. R. LONG.

